# A Spectrum of Intraoperative and Postoperative Complications of Cochlear Implants: A Critical Review

**DOI:** 10.7759/cureus.28151

**Published:** 2022-08-18

**Authors:** Isha Sahai, Benumadhab Ghosh, Ashish Anjankar

**Affiliations:** 1 Otolaryngology, Jawaharlal Nehru Medical College, Datta Meghe Institute of Medical Sciences, Wardha, IND; 2 Biochemistry, Jawaharlal Nehru Medical College, Datta Meghe Institute of Medical Sciences, Wardha, IND

**Keywords:** surgery, complications, pneumocephalus, cholesteatoma, meniere's disease, tinnitus, implantation

## Abstract

A cochlear implant is a neuroprosthetic, electrical device that is developed for the treatment of patients who have sensory hearing loss. It directly stimulates the hearing nerve by bypassing the injured or damaged sensory receptors, the hair cells. This implant is directly placed in the inner ear. It is an electronic device which is proved to be very useful in patients with sensorineural hearing loss. This implant consists of a speech processor (externally present), which takes up the sound; transforms it into digital signals, and then internal components take it to convert it into electrical energy, which stimulates auditory nerves, and the brain perceives it and hears it as a sound. This is one of the most successful surgeries, which happens very frequently nowadays. Although, many complications are mostly associated with this implant. This paper deals with the preoperative, operative, and postoperative complications associated with cochlear implant surgery. That includes tinnitus, Meniere's disease, unilateral hearing loss, musical ear syndrome, infections; flap necrosis, facial nerve palsy, improper electrode placement, magnet displacement; failure and re-implantation, cholesteatoma, and pneumocephalus. These are just a few of the complications; there are much more complications which are associated with cochlear implants.

## Introduction and background

The cochlea is a structure of coiled nature present in the ventral area of the inner ear and is the main organ for perceiving sound. The Organ of Corti lines throughout the spiral of the cochlea, the sensory epithelium, which is highly derived and rigorously patterned which helps in converting stimuli of auditory into impulses of the nerve [1]. A prevalence study conducted in India showed that around 15.14% of the population of rural origin and around 5.9% of the urban population had hearing impairment [2]. There was a study in eastern India to understand the prevalence of profound SNHL (sensorineural hearing loss) in children, which stated around 0.058%. The incidence of sudden sensorineural hearing loss is 520 per 100000 [3]. There are various modalities for the treatment of sensorineural hearing loss, such as the use of hearing aid, gene therapy, and sound therapy. The conventional way is using a hearing aid, which is one of the most common treatments for sensorineural hearing loss. This is convenient and easy for patients of all ages as they just have to wear the hearing aid around their ear to help with their hearing [4]. Gene therapy is an important treatment option for hearing loss [5]. Another way is sound therapy, a recent development in hearing loss treatment. It is conducted alongside medication to improve the adaptability and response to different sounds [2]. 

When a person is unable to hear, cochlear implants (CI) can be used, which are the best solution for the perception of speech for most users [6]. The objective of the implant is to skip the defective surgery organ, i.e., the organ of Corti stimulates the hearing nerve electrically [6]. Patients with complete sensory input loss can perceive speech using this implant input [7]. The treatment choice for rehabilitation in patients with sensorineural deafness is cochlear implantation [8]. It helps restore the lost function of the sensory receptors (hair cells) by converting the acoustic signal into stimulus (electrical), which causes activation of hearing nerve fibers [9]. After electrical stimulation of the auditory nerve, Cochlear implants constitute an interface re-connecting the brain and the speech to be heard by providing information regarding the sound to be perceived [10]. The implants take the sounds present in the environment with the sounds of a person speaking, using microphones, which are integrated within the speech processor. Acoustic signals that the processor records are then transformed into the digital domain for pre-processing. Multiple methods, which are pre-processed, are used nowadays to improve the ratio of signal-to-noise. This ratio is between the speeches received from a target speaker and the noise in the background or the reverberation [11]. It includes directional microphones, which are necessary for filtering and noise reduction, which are single channels. Cochlear implants are the world's most successful and commonly done neuro-sensory prosthesis [12]. It has been the subject of research in recent years. There are strategies for the enhancement of speech and dereverberation [13]. The cochlea is a structure of coiled nature present in the ventral area of the inner ear and is the main organ for perceiving sound. The organ of Corti lines throughout the spiral of the cochlea, sensory epithelium, which is highly derived and rigorously patterned, which helps in converting stimuli auditory into impulses of nerve [14].

Cochlear implant surgery is a routinely done surgery that is performed under general anesthesia. Behind the ear, a small incision is made by the surgeon. The cochlear implant is kept under the skin, and the electrodes are inserted into the inner ear. Tests for measuring the response to the implant are done by a team of surgeons. Further, they would close the incision using disposable stitches. Cochlear implants and hearing aids are two different devices. Amplification of the sound is the main function of a hearing aid. This amplified sound is detected by the ear, which is damaged. Many people recognize warning signals with the help of this device. Implant users can understand other sounds present in the environment. They understand what the other person is saying [15].

Indications of cochlear implants are bilateral (B/L) sensorineural hearing loss (SNHL), B/L deafness (post-lingual), B/L loss of hearing for frequency in higher ranges whilst maintaining frequency of lower ranges, in aids of hearing absence - stimulation of electroacoustic nature and asymmetrical hearing loss with deaf ears having serious tinnitus, which other hearing aids cannot treat, like the bone conduction implant or bi-contralateral routing of signals system (biCROS/CROS), treatment should be done one by one. There are various special cases in which we need fast intervention with implantation [16]. These cases could be meningitis associated with hearing damage, fibrosis found within fluid spaces in the cochlea, bilateral implantation in progressive amblyopia with significant hearing impairment, like Usher syndrome, and implantation which has no upper age limit [16]. The efficiency of the body and systemic complication risks must be checked before performing surgery, especially in elderly patients, where it is important to check for central hearing damage [16]. 

Methodology

**Figure 1:**
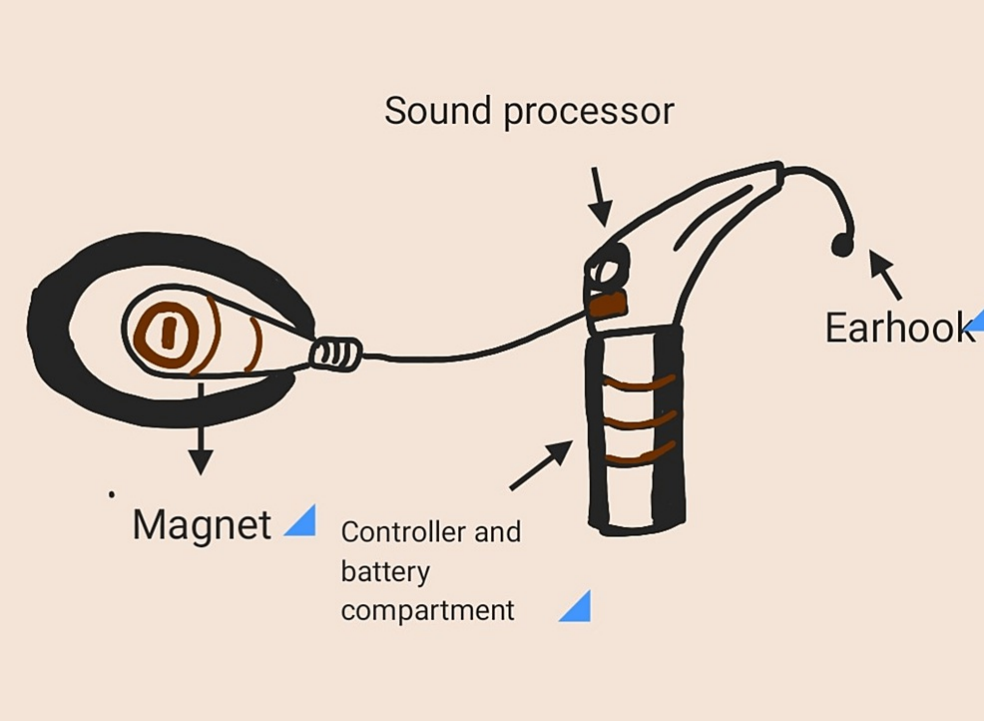
Structure of the implant This figure is the sole creation of the author.

Structural components of an implant are a magnet, controller and battery compartment, sound processor, and ear hook (Figure 1) [17]. 

Mechanism of action

The Cochlear implant takes the sounds present in the environment with the sounds of a person speaking, using microphones that are integrated within the speech processor. Acoustic signals, which are recorded by the processor, are then transformed into the digital domain for pre-processing [10]. 

**Figure 2:**
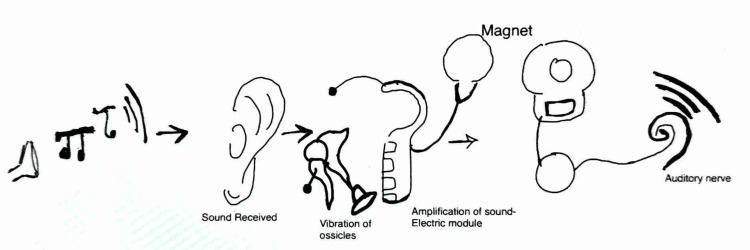
Mechanism of action of the implant The figure is the sole creation of the author.

CI functions by a transduction mechanism in which acoustic energy is converted into an electrical impulse or signal and is used for stimulating the already surviving cells of the spiral ganglion of the 8^th^ cranial nerve (cochlear part) (Figure 2) [17,18].

## Review

Components of a cochlear implant

The implant consists of the following parts: A speech processor, transmitter, receiver, and electrode array (Table 1) [15]. 

**Table 1:** Parts of a cochlear implant

Parts	Functions
Microphone	Captures sound
Speech Processor	Arrangement of the captured sounds
Transmitter and Receiver	Signal received and convert it into electrical impulses
Electrode Array	Impulse collection and sends it to auditory nerve

Cochlear implant surgery has various advantages and disadvantages when performed on patients with complications. These are listed in Table 2 [9].

**Table 2:** A comparative representation of cochlear implant

Technique	Advantages	Disadvantages	Comparison with Hearing Aid
Cochlear Implant	It is the treatment choice for rehabilitation in patients with sensorineural deafness is cochlear implantation [5].	Malfunctioning of the equipment. Perception of the sound from the environment. Perception of the speech when speechreading is used or not used. Psychological issues. Social and lifestyle changes.	Hearing aid: Amplification of sound and stimulation of inner hair cells. Cochlear implant: Works by stimulating the organ of Corti and electrically stimulating the nerve.

Pre-operative indications

Tinnitus is a condition in which the patient experiences a ringing sensation in the ear. Before performing the surgical operation of putting the cochlear implant, tinnitus is a condition that is commonly indicated. There are studies in which it has been found that there are noticeable changes in the perceptivity of tinnitus after a cochlear implant [19]. Stimulation of cochlea by electricity can have the ability to reestablish networks of tonic inhibition and thereby suppresses tinnitus [20]. Cochlear implantation and labyrinthectomy together help in gaining the restoration of binaural hearing sensation. There is a potential vertigo elimination along with tinnitus by using ablation processes under general anesthesia [21]. Labyrinthectomy is not the reason for this reduction; cochlear implantation is because older studies didn't show tinnitus reduction when implant placement is not done, and the labyrinth is removed [22]. Patients with profound bilateral deafness (BD) are prone to suffering from tinnitus, which further leads to psychological comorbidities and makes it more difficult for patients to communicate with people. Tinnitus was reduced by cochlear implantation whilst CI was on rather than off. Implantation seemed to decrease depression/anxiety severity. Before and after the surgery, positive correlations were found between tinnitus and depression/anxiety severity [23].

Meniere’s disease (MD) is another clinical indication for cochlear implantation. It is an inner ear disorder in which certain clinical features have been observed, including loss of hearing, ringing sensation in the ear (tinnitus), and spinning sensation (vertigo). In most cases, it is a gradually progressive condition that has a remarkable impact on the person's social life [24]. The prevalence rate of Meniere’s disease (MD) lies between a range of 3.5 in 100000 to 513 in 100000 [25]. Vestibular dysfunction causing long-term problems is seen in Meniere’s disease undergoing CI patients. In patients with bilateral Meniere's disease (MD), composite and predicted measures in the QoL (quality of life) will help in better management of both vestibular and audiological outcomes [26]. In cases of failure in intratympanic and medical treatment in Meniere's disease (MD), surgical management is endorsed. The Gold standard process of denervation in controlling vertigo attacks is the translabyrinthine vestibular nerve section. However, the residual hearing is sacrificed in this case. No work, to our knowledge, has been published regarding patients who underwent trans-labyrinthine vestibular neurectomy and further CI for Meniere's Disease [27]. In advanced MD cases, CI provides good outcomes concerning performance in speech, implantation age, bi- or unilaterality, older therapeutic intervention, and activity stage of MD [28]. Cochlear implantation investigations in Meniere's have shown success in rehabilitation of the auditory in various studies [29,30].

Apart from these major complications, it is also observed in the pediatric population that the application of CI in unilateral hearing loss is a newly approved treatment modality by FDA (Food and Drug Administration) in 2019 [31]. Studies are conducted that supports the use of CI in patients of AHL (Asymmetric Hearing Loss)/SSD (Single-Sided Deafness). CI is a unique technique for this set of patients because binaural hearing is restored. A review shows major CI improvement in the perception of speech in quiet and noise, tinnitus, localisation of sound and QoL. Also, these show better improvements than older options such as CROS aid or bone conduction device (BCD) [32].

Operative complications

While performing the operative procedures of a cochlear implant, multiple complications can be observed. Anatomical malformations of the middle ear can cause complexity in cochlear implant surgeries. These abnormalities could be inner ear malformations which include cochlear aplasia, hypoplasia of the cochlea, incomplete partition with a prevalence of 40%-100%, hypoplastic mastoid with 55.2% prevalence, aberrant facial nerve course with 36% prevalence rate, aplastic round window with 71% prevalence and malformation of the vestibuli [33]. These abnormalities may resist the approach to the round window using the conventional method to reach the facial recess for putting in the implant [33].

It has been estimated that around 46.58% is the actual exposure of the facial nerve in iatrogenic (physician-induced) dehiscence [34]. The anatomical pathway and location of the facial nerve can be different and hence can lead to exposure of the facial nerve during surgical operations, thus leading to its damage. Amongst this, only 2.1% is the actual incidence of facial nerve palsy in post-operative conditions. This number has decreased to 0.72% in cases with no injury to the facial nerve sheath [34]. Hence facial nerve palsy is one of the common complications observed during the procedure of putting on an implant. Going by the anatomical location of the facial nerve, there's a high risk of injury to this nerve while performing cochlear implant surgery, specifically during posterior tympanotomy [35]. 

Flap necrosis is another major surgical complication observed during cochlear implant surgery, which has a rate of 1.4% [36]. It causes the death of tissue of the flap, which is placed over the stimulator coil (receiver part). There is a case study of a woman who is 55 years old and has undergone cochlear implantation, where the patient presents with bluish-colored necrosis of the skin, which occurred due to marking over the bone [37]. Skin flap necrosis is found to be one of the commonest problems related to the use of implants [38]. 

Another complication during surgery is improper placement of the electrode. It is one of the major complications during cochlear implant surgery, with a rate of 3.8% [39]. While inserting the electrode, there is a high chance of a scalar translocation. In this translocation, the electrode reaches scala vestibuli from scala tympani via the basilar membrane [39]. Since there is a scalar translocation of the electrode, an increased risk of damage to the structures present in the inner ear like a lateral wall of the cochlea, basilar membrane, spiral ligament, spiral osseous lamina, and near to it while performing cochlear implant surgery has been noted [39]. 

There could be another type of complication seen in cochlear implants, magnet displacement [40]. A study was conducted on 6469 patients on the magnet displacement- receiver migration in which it was found that magnet displacement has been reported in around 1.3% of patients, while receiver migration has been found in around 0.1% of cases [41]. It has been seen that the main cause for magnet dislocation was found to be the rotational force resulting from the torque experienced inside the magnet bore [42]. 

Postoperative complications

In virtue of failure and re-implantation of the implant, the process of CI is a secure rehab surgical intervention for hearing with a low rate of complication. There are minor postoperative complications that rightly cause infection in children, such as acute otitis media. In the case of adults, it is cochleovestibular, i.e., tinnitus and vertigo. Major complications include re-implantation after due surgery or in case of failure of the device [43]. 

Other operative complications of cochlear implants may include Gusher syndrome, which means profuse amounts of CSF (cerebrospinal fluid) observed while performing cochleostomy in CI. As per a study, which included 415 patients, it was found that 39 gushers were observed to have an incidence rate of about 9.39% [44]. This syndrome was earlier known as perilymphatic gusher which was observed in patients having broad IAM (internal acoustic meatus) or LVAS (large vestibular aqueduct syndrome) while performing stapedectomy(h). Infections are another complication that is observed in post-operative conditions. The incision made for the cochlear implant surgery and its relation to the post-operative wound infections were observed and invested by Ray et al. He observed that there was a 2.4% prevalence of infections and pores at the surgical site for those implants which had larger incisions. Also, a 1.1% incidence of skin infections was observed in small incision cochlear implant surgery [45]. Anatomical abnormalities could be another complication. Cochlear implant surgery gets more complex and difficult for the surgeon if inner ear malformations are present in the patient. In some cases, it has been observed that CSF gushes in after the opening of the cochlea during surgery. Studies have reported that the incidence of these gushes ranges between 0.4% and 5.58% [46]. These limitations can be overcome by attending to the various details of the surgery, which can be extremely helpful and necessary. These surgical details are conical external base arrays, small cochleostomy, and complete intraoperative sealing of the internal ear. 

Apart from these significant postoperative complications, Injury to the facial nerve has been another postoperative complication. The prevalence rate of transient facial nerve palsy in cochlear implant patients has been observed to be around 1.4% [46]. Postoperative facial nerve weakness could be a minor complication that has been observed. This weakness could be due to edema or due to herpes virus reactivation. It is seen not only after cochlear implantation but also while performing tympanomastoid procedures [46]. 

Another major complication that is also followed by an implant surgery is musical ear syndrome (MES). This is a syndrome in which there is the perception of musical sensations in the auditory system without any external stimulus. It is described as a rare situation called musical ear syndrome [47]. Prevalence of this condition was seen higher in patients who have undergone cochlear implant surgery. A study showed that around 22% of patients who had their cochlear implant surgery developed musical ear syndrome. This condition was seen to be more associated with younger aged patients [48]. 

Pneumocephalus is another rare postoperative condition that causes seizures and is rarely seen to happen weeks after post-surgery. Only five cases for this have been reported as searched on PubMed as on 8^th^ August 2022 [49]. They are manifestations occurring in nerves with a high potential to harm the life of a patient, so the necessity arises in correct comprehension to manage the conditions. It should be taken care of pneumocephalus, whether bone or mastoid defects are doubted in surgery, as features of neurology in a patient with CI. Management is primarily conservative in nature, and clinical observations and radiological imaging in managing pneumocephalus. Surgery is done in tension pneumocephalus and symptomatic patients as a treatment [49]. 

The occurrence of cholesteatoma (0.95%) has also been observed in CI patients [50]. There is a 0.25% incidence of congenital cholesteatoma in CI patients. The incidence is higher than expected for this rare condition [51].

**Figure 3:**
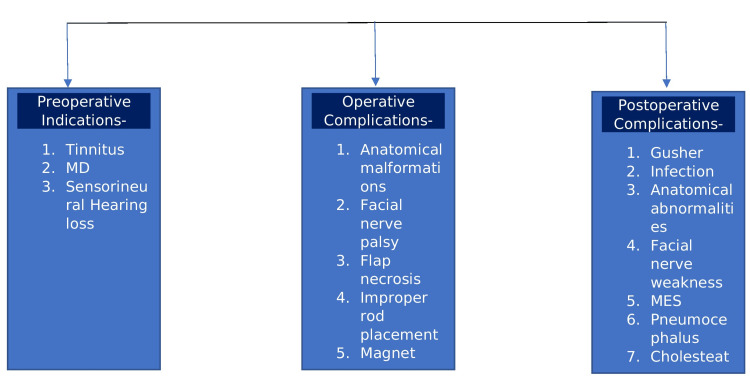
Show Illustration of complications This flowchart is the sole creation of the author.

The critically classified surgical complications, as well as the indications, since pre-operative indications, operative, and postoperative complications are illustrated in Figure 3. 

## Conclusions

Cochlear implants are one of the most effective treatment options for the rehabilitation of patients who have sensorineural hearing loss. Our study included preoperative indications and operative and postoperative complications in which it was noted that there are multiple complications with high prevalence rates, which are associated with cochlear implants. These complications could range from simple infections like otitis media, incision infections, or wound infections; to facial nerve palsies. We have also included certain rare complications in our study, which include pneumocephalus, anatomical malformations of the ear, electrode displacements, and implant failure.

Proper indications for the use of implants, highly sensitive imaging modalities, and thorough knowledge about the anatomy of the ear and nearby structures may prove a boon in reducing complications.
